# Parenteral Cu Supplementation of Late-Gestating and Lactating Iberian Red Deer Hinds Fed a Balanced Diet Reduces Somatic Cell Count and Modifies Mineral Profile of Milk

**DOI:** 10.3390/ani10010083

**Published:** 2020-01-03

**Authors:** Martina Pérez Serrano, Andrés José García, Tomás Landete-Castillejos, Jamil Cappelli, José Ángel Gómez, Francisco Hidalgo, Laureano Gallego

**Affiliations:** 1 Animal Science Techniques Applied to Wildlife Management Research Group, Instituto de Investigación en Recursos Cinegéticos, Universidad de Castilla-La Mancha, Campus Universitario sn, 02071 Albacete, Spain; AndresJose.Garcia@uclm.es (A.J.G.); Tomas.Landete@uclm.es (T.L.-C.); Jamil.Cappelli@uclm.es (J.C.); Laureano.Gallego@uclm.es (L.G.); 2 Sección de Recursos Cinegéticos y Ganaderos, Instituto de Desarrollo Regional, Universidad de Castilla-La Mancha, Campus Universitario sn, 02071 Albacete, Spain; jagomezn@jccm.es (J.Á.G.); hidalgo.paco@gmail.com (F.H.); 3 Departamento de Ciencia y Tecnología Agroforestal y Genética, Escuela Técnica Superior de Ingenieros Agrónomos y Montes, Universidad de Castilla-La Mancha, Campus Universitario sn, 02071 Albacete, Spain

**Keywords:** Cu supply, Iberian hinds, lactation stage, milk nutrient, milk production, offspring growth

## Abstract

**Simple Summary:**

The benefits of Cu supplementation have been reported for male deer and cows. In deer, supplementation with this mineral is frequent in males even in animals receiving balanced diets. The benefits of Cu supplementation for antler cortical thickness in adult deer and for deer meat characteristics have also been demonstrated. Since Cu supplementation of cows has positive effects to achieve optimal offspring growth, it could be interesting to assess such effects for hinds since this mineral is transported across the placenta and has an important role in animal growth. Our results in lactating hinds showed that Cu supplementation decreased the number of somatic cells, improving the health quality of the milk and indicating a lower risk of mastitis. It also increased Ca and K content of milk, although not that of Cu. This is the first study demonstrating that Cu supplementation during late-gestation and lactation in hinds fed a balanced diet could be a good strategy to reduce somatic cell count (improving the health quality of milk) and modify the mineral profile of milk increasing its Ca content.

**Abstract:**

This study describes the effects that Cu supplementation of late-gestating and lactating females (hinds) of Iberian red deer fed a balanced diet have on milk production, composition, and somatic cell count (SCC). Experimental hinds (*n* = 9) were subcutaneously injected every 42 days with Cu (0.83 mg Cu/kg body weight) from day 202 of gestation until the end of lactation (week 18). Control hinds (*n* = 8) were injected with a physiological saline solution with the same volume and at the same frequency as the experimental group. Copper supplementation decreased the SCC from 1.64 to 1.36 log 10/mL (*p* = 0.003) and modified the milk mineral profile. In particular, milk from hinds supplemented with Cu had more Ca (*p* = 0.02), Mg (*p* = 0.06), and K (*p* = 0.03) than milk from control hinds. However, Cu supplementation did not influence the Cu content of milk. Therefore, it can be concluded that Cu supplementation of hinds fed a balanced diet from late-gestation to the end of lactation could be a good strategy to reduce SCC and modify the mineral profile of milk increasing its Ca content.

## 1. Introduction

Insufficient Cu constitutes one of the most common and severe trace mineral deficiencies in deer [1]. These animals frequently require Cu supplementation. However, Cu supplementation presents benefits even when deer are fed balanced diets. Thus, Gambín et al. [2] found that supplementation with Cu increased antler cortical thickness in adult deer. This may improve the trophy value of antlers. Should Cu supplementation have similar effects on bones as those observed in antlers this may bring potential benefits for bones in elderly humans. In addition, supplementation with Cu could improve deer meat characteristics [3].

Regarding females, the importance of Cu supplementation has been extensively studied for cows. In fact, trace mineral supplementation (including Cu) of late-gestating cows is essential to achieve optimal offspring growth even when animals ingest balanced diets and do not present mineral deficiencies [4]. In this sense, Naylor et al. [5] studied the effects of Cu supplementation levels (40 vs. 5.5 mg of Cu per kg of dry matter (DM) of diet) on beef cows without Cu deficit during the last trimester of pregnancy. These authors observed an improvement of the average daily gain of offspring with the higher level of Cu. Moreover, Cu supplementation might be useful during lactation of hinds as it has been observed in cows fed balanced diets to modify production [6] and composition [7] of milk and to reduce its somatic cell count (SCC) [8]. However, the impact of Cu supplementation during gestation and lactation has not been studied previously for hinds fed a balanced diet.

Parenteral mineral supplementation is a common practice in deer [2,9,10]. Although this technique requires a more laborious handling than minerals added to feed, it ensures that the same quantity of the mineral is supplied for all animals, and also that minerals supplemented bypass the gastrointestinal tract, thus avoiding interactions with other minerals leading to secondary deficiencies. These secondary deficiencies are caused by the high content of mineral antagonists in the diet (such as S, Mo, Fe, and Mn for the case of Cu) that may decrease the absorption of the supplemented mineral [11] in animals without deficiencies.

Since Cu is transported across the placenta mainly during the end of gestation [12] and plays an important role on growth [13], it is expected that Cu supplementation of late-gestating hinds without Cu deficit might improve the growth rate of calves. Characterization of deer milk is very interesting since several anecdotal reports suggest the potential health effects for deer milk and their fermented products, but scientific evidence to support this is still under investigation. The aim of this manuscript was to study the effects of parenteral Cu supplementation from day 202 of gestation (total gestation average duration of 233–234 days) to 18 weeks of lactation of red deer hinds fed a balanced diet on offspring growth performance from birth to weaning, milk production, milk composition, and SCC.

## 2. Materials and Methods

### 2.1. Ethical Statement

This study was carried out in accordance with the Spanish legislation for the use of animals in research [14] and the approval of the Ethical Committee in Animal Experimentation from the University of Castilla-La Mancha (Permit Number: 1002.04). Handling and sampling were designed to minimize stress and health risks. Hinds and calves were examined daily by farm personnel and weekly by an experienced veterinarian.

### 2.2. Animals and Management

Copper supplemented (*n* = 9) and control (*n* = 8) Iberian red hinds were reared in captivity conditions and fed simulating the feeding system of deer farms using the same feeding program described by Serrano et al. [10]. Briefly, hinds were kept in a 5000 m^2^ open-door enclosure on an irrigated mixed pasture. To optimize management in the experimental farm, the feed (including the concentrate and the ration) was homogenized and reduced to a mixture in a tractor-driven commercial mixer. The feeding program was common for all animals and met or exceeded the nutrient requirements for late pregnancy and lactation of hinds recommended by the National Research Council [15]. Copper contribution was 28.0 mg per kg of diet and 11.8 mg per kg of ration. Feed and water were offered ad libitum throughout the trial. Calves had free access to concentrate and ration of hinds.

### 2.3. Experimental Design

Experimental hinds were subcutaneous injected every 42 days with 0.83 mg of Cu per kg body weight (BW; 1 cm^3^ per 30 kg live BW) from day 202 of gestation to day 126 (week 18) of lactation (standard lactation period in natural conditions of hinds) using Glypondin (König S.A., Buenos Aires, Argentina) as previously used by Gambín et al. [2] for deer. The control group was injected with a physiological saline solution with the same volume and at the same frequency as the experimental group.

### 2.4. Measurements, Sampling, and Analyses

The BW and body condition score (BCS) of hinds were obtained at the beginning of the trial (first Cu injection at day 202 of gestation as average), 3.5 days before, and 3.5 days after calving (±1 day in both cases). Moreover, hinds were individually weighed at weeks 2, 4, 6, 10, 14, and 18 of lactation. The same electronic balance (±50 g. model KC 300 S, Mettler-Toledo S.A.E., L’Hospitalet de Llobregat, Barcelona, Spain) was used in all cases. The BCS of hinds was measured according to Carrión et al. [16]. At the beginning of the trial, hinds were assigned to the experimental groups so that the average (± standard error of mean, SEM) age (9.8 ± 1.68 vs. 10.8 ± 1.65 year), the average BW (105.2 ± 2.25 vs. 110.3 ± 2.72 kg), and the average BCS (3.64 ± 0.073 vs. 3.78 ± 0.137) were statistically the same for both groups (Cu supplemented and control groups, respectively).

The mean calving date was 14 May ± 3.1 days in the experimental group (4 male and 5 female calves) and 17 May ± 2.7 days in the control group (4 male and 4 female calves). Calves were individually weighed within the first 12 h of life (birth BW) and weekly up to week 18 (weaning BW enforced by physical separation) of lactation using the same electronic balance used for their mothers (± 50 g). The relative BW gain of calves from birth to weaning was calculated as indicated by Landete-Castillejos et al. [17].

During the days of milking at weeks 2, 4, 6, 10, 14, and 18 of lactation, hinds were separated from their calves for 6 h (08:00 to 14:00 h) in a handling facility [18,19]. Milking was conducted using a machine milking set-up 50/50 massage/milking ratio and 44 kPa of vacuum following the procedure described for hinds by Serrano et al. [10]. Briefly, a low-dose of anesthesia was injected in the right jugular vein using 0.5 mg per kg BW of xylazine (Seton 2%, Calier, Barcelona, Spain) and 1 mg per kg BW of ketamine (Imalgene 100, Merial, Lyon, France) before milking. After inducing anesthesia and 1 min before milking was started, oxytocin (10 I.U.; Facilpart, Laboratorios Syva, León, Spain) was injected in the right jugular vein. When milking was finished, anesthesia was reversed with a yohimbine injection (0.25 mg per kg BW, Sigma Chemical Co., St. Louis, MO, USA). The daily milk production (DMP, mL/day) was calculated by multiplying the milk production collected per milking by four [18,19] and these data were used to calculate the total milk yield (TMY).

Two milk samples (30 mL each one) were collected from each hind to assess milk composition (fat, protein, lactose, and DM concentrations). Therefore, milk composition was obtained as the mean of the two aliquot replicates from each milking. Samples were diluted by 50% with distilled water to adjust their composition to the calibration range of the automatic milk analyzer (Milkoscan series 4000, FOSS Analytical A/S, Hillerød, Denmark). Data obtained for milk nutrient composition were used to calculate protein:fat ratio (PFR) and total fat yield (TFY), total protein yield (TPY), total lactose yield (TLY), and total DM yield (TDMY) during lactation. The gross energy content of fresh milk (GEFM) was calculated as suggested by Landete-Castillejos et al. [20]. In addition, the total daily gross energy (TDGE) produced in milk was calculated multiplying the GEFM by the DMP as indicated by Serrano et al. [10]. The SCC was analyzed using a Fossomatic 5000 (Foss Electric, Hilleroed, Denmark) based on flow cytometry technology that counts somatic cells in compliance with the International Organization for Standardization/International Dairy Federation and National Conference on Interstate Milk Shipments.

An aliquot of each sample (2 mL) collected at 2, 4, 14, and 18 weeks of lactation was frozen (−20 °C) for subsequent mineral analyses. To assess Cu content in serum, blood samples (5 cc) were taken from resting hinds after an overnight of fasting just before the first injection with Cu (basal level) and at 7 days of study. Blood samples were drawn, without sedation, by jugular venipuncture (Vacutest Clot activators, Kima S.A., Arzergrande, Italy) and were coagulated in the tubes. Serum was separated using a centrifugal separator (1372× *g* at 4 °C for 15 min) and samples were frozen (−20 °C) for subsequent analysis of Cu content. Considering animal welfare, no further measurements of serum Cu level were made throughout the study to avoid subjecting more punctures than strictly necessary to experimental animals.

Blood samples were drawn, without sedation, by jugular venipuncture (Vacutest Clot activators, Kima S.A., Arzergrande, Italy) and were coagulated in the tubes (5 cc). Serum was separated using a centrifugal separator (1372× *g* at 4 °C for 15 min) and samples were frozen (−20 °C) for subsequent analyses of Cu content. Mineral content of milk and Cu content of concentrate, ration and serum were analyzed by inductively coupled optical emission spectrometry (ICP 6500 DUO Spectrometer/IRIS INTR.EPID II XDL; Thermo Fisher Scientific Inc., Waltham, MA, USA).

### 2.5. Statistical Analyses

All data were normally distributed according to the Shapiro_Wilk test. The effects of Cu supplementation on BW and BCS (hinds and calves), yields (TMY, TFY, TPY, TLY, TDMY), and serum Cu content data were analyzed using a general linear model (GLM). The experimental units were not blocked by calf sex because hinds were assigned to groups at gestation stage before knowing the sex of their fetus. Then, the interaction between treatment and sex was not tested. Calf birth BW was used as a covariate to analyze BW at weaning, but it was not significant (*p* > 0.10) and it was removed from the analyses. The Cu content of serum samples collected at the beginning of the trial (basal level) was used as a covariate to analyze the Cu content of serum at 7 days after the first Cu injection. In addition, a GLM analysis was performed to study the sex effects of offspring in milk production and milk composition. In this case, global data (including those from Cu supplemented and control groups) were used because no significant interactions between sex and Cu supplementation were detected for any traits.

On the other hand, the effects of Cu supplementation, hind within treatment, lactation week, and Cu supplementation × lactation week interaction were tested for BW of hind and calves throughout lactation, DMP, milk composition (fat, protein, PFR, lactose, DM and mineral content), GEFM, TDGE, and SCC using a MIXED procedure. Based on current results, the effects of Cu supplementation were independent of the lactation week. The effects of Cu supplementation and lactation week were analyzed in an independent way. A Tukey test was used to make pairwise comparison among means when the model was significant for the lactation week. In addition, relations between traits were analyzed using bilateral Pearson correlations for pooled, Cu supplemented, and control groups.

In all cases, analyses were carried out with SPSS version 19 (SPSS Inc., Chicago, IL, USA) and the replicate was the animal (*n* = 9 for Cu supplemented group and *n* = 8 for control group). Data in tables are presented as means for all traits except for Cu serum content at 7 days of trial (basal level of Cu as covariate) which is presented as least squares means.

## 3. Results

No significant interactions between Cu supplementation and lactation week were detected. Therefore, only main effects are shown.

### 3.1. Effects of Cu Supplementation on Hind and Calf Traits

Copper supplementation did not influence BW or BCS of hinds at any stage, nor BW of calves at birth or weaning (Table 1). In addition, when the variability in calf birth BW was considered expressing BW gains until weaning as a percent relative to calf birth BW (calf relative BW gain), calves from hinds that were supplemented with Cu showed a similar BW gain from birth to weaning at 18 weeks of age compared to calves from control hinds (*p* > 0.10). Moreover, no differences were observed between groups for yields, DMP, milk content of fat, protein, lactose and DM, PFR, GEFM or TDGE (Table 2).

As expected, no differences (mean ± SEM) were observed for the Cu content of serum between Cu injected and control groups before supplementation started (0.33 ± 0.104 and 0.39 ± 0.048 ppm, respectively). Average Cu content after 7 days of supplementation was 0.68 ± 0.066 and 0.40 ± 0.099 ppm for Cu injected and control groups, respectively. The increase in the content of Cu in serum from the basal level (pre-injection) to the level after 7 days of treatment showed an increase in animals that were injected with Cu, whereas it remained approximately the same level with just a small increase in animals injected with the saline control (increase pre-post treatment 0.35 ± 0.104 in Cu injected group vs. 0.07 ± 0.087 ppm in saline injected control). However, despite that the increase in the Cu injected group was five times that of saline control, the difference did not reach statistical significance (*p* = 0.101).

Regarding milk mineral composition, supplementation with Cu did not influence the content of this mineral in milk but increased its content of Ca (*p* = 0.02), Mg (*p* = 0.06), K (*p* = 0.03), and Sr (*p* = 0.001) (Table 2). In addition, Cu supplementation decreased the SCC from 1.64 to 1.36 log 10/mL (*p* = 0.003).

### 3.2. Effects of Lactation Week on Hind and Calf Traits

Hind BW did not change throughout lactation (Table 3). However, DMP decreased (*p* < 0.001) from 3140 mL per day at week 2 to 1582 mL per day at week 18. These decreases in milk production did not remain stable during lactation and were highest at the beginning (13.5%, week 2 to 6) and end of the study period (25.0%, week 14 to 18) than between weeks 10 and 14 of lactation (less than 1%).

Fat, protein, lactose, and DM contents of milk increased (*p* < 0.001) with lactation week. The PFR was affected (*p* < 0.001) by the lactation stage and protein was substituted by fat over the lactation period. On the other hand, the GEFM (1587 kcal/kg as average) increased (*p* < 0.001) and the TDGE (3774 kcal/day as average) decreased (*p =* 0.005) throughout the lactation. The SCC averaged 1.48 log 10/mL over the 18-week period and decreased (*p* < 0.05) with lactation stage from weeks 2, 4, and 6 to weeks 10 and 14 with values for week 18 being intermediate. In contrast, P (*p* = 0.08), Mg (*p* = 0.05), and Zn (*p* = 0.007) contents increased between weeks 2 and 18 of lactation (Table 4).

### 3.3. Effects of Calf Sex on Production and Composition of Milk

Milk for females had greater (*p* < 0.001) content of fat (10.6% ± 0.25 vs. 9.1% ± 0.22) and DM (24.3% ± 0.28 vs. 22.8% ± 0.22) than milk for males. However, sex of offspring did not influence on DMP, protein, lactose, and mineral content of milk. Finally, no differences (*p* = 0.11) between sexes were observed for BW at birth, although males tended (*p* = 0.06) to be heavier than females at weaning (54.9 ± 2.98 vs. 48.8 ± 0.92 kg).

### 3.4. Correlation Coefficients between Variables

The SCC was not correlated with DMP, fat or protein contents of milk (data not shown). However, a negative correlation (*p* < 0.05) was observed between the SCC and the lactose content of milk (*r* = −0.533 and −0.932 for pooled and control groups, respectively). In addition, SCC correlated positively with milk contents of Na (*r* = 0.481 and *p* < 0.10 and *r* = 0.723 and *p* < 0.05 for pooled and Cu supplemented groups, respectively), Mg (*r* = 0.756 and *p* < 0.05 for control data), and Mn (*r* = 0.677 and *p* < 0.10 for control data).

## 4. Discussion

Mineral supplementation in deer is common mainly in situations where there are deficiencies. However, available information describing the effects of supplying minerals that are important for the nutrition of this species (such as Mn or Cu) in animals fed balanced diets is very scarce. Cappelli et al. [9] concluded that Mn supplementation in deer fed a balanced diet increased impact energy and contents in minerals of antler bone tissue. Recently, Serrano et al. [10] concluded that parenteral Mn supplementation of late-gestating and lactating hinds is recommended, even when they are fed a balanced diet, to increase milk production and content of fat, Ca, P, and Mn. This studied proved that parenteral Cu supplementation reduced SCC improving udder health and modifying the milk mineral profile which increased its content of Ca.

Current results showed that no Cu deficiencies were detected one week after the first Cu injection even for the control hinds (0.60 ± 0.079 and 0.52 ± 0.104 ppm for Cu supplemented and control groups, respectively) according to the mentioned criteria set by Wilson and Grace [21]. These authors proposed a reference criterion for deer serum Cu content considering serum Cu values lower than 0.32 ppm as deficiency and higher than 0.50 ppm as adequate. In fact, serum Cu levels were adequate from the beginning of the study (basal levels of 0.36 ppm as average for both groups). Therefore, the current study is the first one that investigates the effect of Cu supplementation in hinds without Cu deficiencies.

Trace minerals are essential for fetal development [22] as the fetus depends completely on the mother for the proper supply of these elements. During gestation, Cu concentration increases progressively in the fetal liver and decreases in the maternal liver [23]. This indicates an increase in Cu requirements by the mother during gestation, mainly during her last month. If maternal mineral supply is inadequate, fetal development and postnatal performance might be impaired [4]. Since Cu is transported across the placenta [24] mainly during the end of gestation [12], Cu supplementation of hinds in mid-to late pregnancy is the most effective way to prevent deficiency of this mineral in the progeny [25]. In fact, hinds with a high concentration of Cu on serum and liver will give birth to fawns with a high serum and liver Cu concentration that remains high until weaning [26]. Some positive effect should be expected with Cu supplementation of gestating hinds for the birth BW of offspring since this mineral plays an important role on growth as has been previously observed for cows without Cu deficiencies [4]. However, current results showed that supplementation of late-gestating hinds with Cu did not affect BW of calves at birth (nor weaning). The cause of discrepancies among authors might be because Cu supplementation of breeding animals fed a balanced diet could improve the offspring growth rate on cows but not in hinds. Therefore, Cu supplementation of hinds may only be effective to improve offspring growth rate when hinds are deficient in Cu.

In the current study, no effects of Cu supplementation were observed for DMP, milk composition, and total nutrient production. No studies about the influence of parenteral Cu supply of hinds on lactating traits are available for deer. However, results about the effects of Cu supplementation are contradictory for dairy cows [7,27,28]. For instance, Sinclair et al. [7] found that BW gain and milk fat content increased and TMY decreased when lactating cows fed a balanced diet were supplemented with organic Cu. Also, Rabiee et al. [27] found that supplementation with organic trace minerals (including Cu) increased DMP by 0.93 kg, milk fat by 0.04 kg, and milk protein by 0.03 kg in lactating dairy cows fed a balanced diet. However, Engle et al. [28] observed that DM intake, average DMP, milk lipid, protein, and SCC were similar across treatments (control without supplemental Cu vs. 10 mg of Cu/kg of DM from CuSO_4_ vs. 40 mg of Cu/kg of DM from CuSO_4_). Thus, Cu supplementation has shown to have different effects in cows depending on the study and seems to differ from our study presented here. Why we did not find any effect on TMY or that of main milk nutrients may be because deer metabolize Cu in a different way to other ruminants [29]. Therefore, although not highly likely, Cu effects may be different in deer and cows. Secondly, the effects of Cu supplementation of lactating hinds on milk production and composition could depend on some factors such as the dose of Cu supplied, the serum level of Cu of hinds at the beginning of supplementation, the Cu supplementation strategy used or the dietary Cu content [24]. In the current study, the dose of Cu used for supplementation was relatively small, because excess of Cu is toxic for some ruminants, including sheep [30] and deer [31]. In fact, the highest values observed for Cu serum 7 days after the first Cu injection (0.68 and 0.40 mg per kg for Cu supplemented and control hinds, respectively) were below the maximum level of 1.7 mg per kg suggested by Laven and Wilson [32] for toxic effects in red deer. Therefore, it is recommended to carry out new studies to test the effects of parenteral Cu supplementation on milk production and milk composition using higher doses than the one used in the current trial.

The average milk Cu content was 0.17 mg/kg (equivalent to 2.7 μmol/L), a similar value to the range from 2.8 to 3.2 μmol/L reported by Grace and Wilson [1] for deer milk. As expected, Cu supplementation did not influence the milk Cu content because it is known that the secretion of this mineral in milk is reduced when the dietary Cu supply is inadequate [33], but it does not increase by supplementing a diet with an adequate level of Cu [11]. Current results agree with those from previous studies conducted with cows where Cu parenteral supply (at different doses) did not produce toxic effects by the increase of milk Cu content [6]. In fact, mammals could protect their offspring and regulate the Cu content in their milk, even if their Cu supply was far too high. However, curiously, milk from hinds supplemented with Cu presented a higher content of Ca than milk from control hinds. Unfortunately, the authors have not found any study about the effects of Cu supplementation of lactating hinds on the mineral profile of milk from deer fed a balanced diet to compare with current results.

In the current study, average values for SCC were 1.36 log 10/mL for milk from Cu supplemented group and 1.63 log 10/mL for milk from the control group; both values were lower than those reported by Pereira [34] for deer milk (4.93 log 10/mL as average for complete lactation). One of the most interesting results of this study regards the positive effects of Cu supplementation reducing the SCC. In addition, a positive correlation between SCC and Na content of milk was detected in the current study. These results agree with those previously observed for cows [35,36]. For example, Summer et al. [35] found higher contents of Na in milk with more than 5.6021 log 10/mL than in milk with less than 5.6021 log 10/mL. Recently, in a review about milk somatic cells and factors influencing their release in dairy animals, Alhussien and Dang [37] concluded that Na is found in high quantity in blood leaked into the milk and increased above normal concentration when subclinical mastitis appeared.

The amount of SCC in milk is an important indicator of udder health. Therefore, based on results obtained for SCC, a lower incidence of mastitis would be expected for Cu supplemented hinds as previously observed for cows [8] by the influence of Cu on immune functions, increasing the phagocytic and killing capacity of neutrophils [38]. There are not reference values for hinds that correlate mastitis incidence with SCC. However, hinds are milked to obtain the milk that is used for cheese production and for other innovative applications in the cosmetic industry. In fact, recent studies have assessed technological variables of red deer milk concluding a good aptitude of this milk for the production of dairy products [39]. Therefore, it is necessary to determine the SCC associated with mastitis in deer. Provided that the effect of Cu was confirmed in other studies in a wider array of herd conditions (different climates and pastures, for example), parenteral Cu supplementation could be used to reduce the SCC of deer milk taking account that Cu supplementation (performed as in the current trial) costs only $4 per animal. It should be noted that some additional factors not included in this cost (i.e., the amount of labor associated with this type of activity, the associated stress of handling, and its impact on production) should be evaluated to determine the profitability of Cu supplementation on gestating and lactating deer to reduce the SCC.

Regarding the lactation stage, results obtained in the current study were similar to those obtained previously in deer by our own research group [10,18,40] and by other groups [41,42]. The information available about the evolution of SCC in deer milk with lactation week is very scarce. Current results showed a decrease of SCC throughout lactation while Pereira [34] observed that SCC decreased between weeks 2 and 10 increasing from week 10 to week 18 of lactation. Therefore, further studies are needed to characterize the evolution of SCC in deer milk throughout lactation.

In summary, Cu supplementation reduced the SCC, improving udder health, and modified the milk mineral profile, increasing its content of Ca. However, more studies are necessary about the influence of mineral nutrition on production and composition of milk produced by deer fed under a balanced diet.

## Figures and Tables

**Table 1:** Effects of parenteral Cu supplementation ^1^ of late-gestating and lactating Iberian red deer hinds fed a balanced diet on body weight (BW) of hind and offspring and body condition score (BCS) of hind and total yields. Data are means ± standard error of mean ^2^.

Item	Cu Supplementation	Control	*p*
Hind BW (kg)			
First Cu injection ^3^	105.2 ± 2.25	110.3 ± 2.72	0.17
Before calving ^4^	120.9 ± 2.09	125.5 ± 2.95	0.21
BW change ^5^	15.7 ± 1.22	15.3 ± 0.59	0.79
After calving ^4^	106.3 ± 1.98	110.8 ± 3.99	0.31
BW change ^6^	−14.6 ± 1.03	−14.7 ± 1.30	0.94
Hind BCS			
First Cu injection ^3^	3.64 ± 0.073	3.78 ± 0.137	0.36
Before calving ^4^	3.94 ± 0.037	3.97 ± 0.099	0.81
BCS change ^5^	0.31 ± 0.056	0.19 ± 0.078	0.23
After calving ^4^	3.86 ± 0.061	3.97 ± 0.137	0.47
BCS change ^6^	−0.08 ± 0.059	0.00 ± 0.047	0.30
Offspring BW (kg)			
Birth	8.08 ± 0.410	8.55 ± 0.471	0.46
Weaning	51.7 ± 1.13	51.7 ± 3.33	0.99
BW change ^7^	43.6 ± 1.13	43.1 ± 3.24	0.88
Offspring relative BW gain (%) ^8^	553.7 ± 37.36	514.1 ± 43.76	0.50
Total yields			
Milk (L)	290.8 ± 22.64	315.7 ± 27.9	0.50
Fat (kg)	29.8 ± 2.53	31.0 ± 2.35	0.73
Protein (kg)	21.1 ± 1.73	22.5 ± 1.98	0.59
Lactose (kg)	13.2 ± 1.10	14.0 ± 1.27	0.66
Dry matter (kg)	71.1 ± 5.67	75.5 ± 6.14	0.61

^1^ Provided by subcutaneous injections containing 0.83 mg of Cu per kg BW. ^2^ The experimental unit was the animal (*n* = 9 for Cu supplemented group and *n* = 8 for control group). In addition, fat, protein, lactose, and dry matter concentrations were computed as the mean of the two aliquot replicates from each milking. ^3^ At the beginning of the trial at day 202 of gestation. ^4^ Measures 3.5 days before and 3.5 days after calving (±1 day in both cases). ^5^ Difference between measures collected before calving and the first Cu injection. ^6^ Difference between measures collected after and before of calving. ^7^ From birth to weaning. ^8^ Calves BW gains from birth to weaning expressed as a percent relative to calf birth BW.

**Table 2:** Effects of parenteral Cu supplementation ^1^ of late-gestating and lactating Iberian red deer hinds fed a balanced diet on body weight (BW) of hind and offspring throughout lactation, daily milk production (DMP), milk composition, gross energy content of fresh milk (GEFM), total daily gross energy content (TDGE), and somatic cell count (SCC). Data are means ± standard error of mean ^2^.

Item	Cu Supplementation	Control	*p* ^3^
Hind BW (kg)	107.3 ± 0.96	109.2 ± 1.44	0.28
Offspring BW (kg)	31.0 ± 1.85	31.9 ± 2.02	0.08
DMP (mL/day)	2303 ± 108.6	2535 ± 159.7	0.49
Milk composition			
Fat (%)	10.0 ± 0.29	9.8 ± 0.30	0.36
Protein (%)	7.0 ± 0.07	7.0 ± 0.09	0.41
Protein:fat ratio	0.72 ± 0.018	0.73 ± 0.018	0.56
Lactose (%)	4.4 ± 0.05	4.3 ± 0.07	0.23
Dry matter (%)	23.7 ± 0.30	23.4 ± 0.33	0.16
Macro minerals (%)			
Ca	0.28 ± 0.013	0.24 ± 0.011	0.02
P	0.21 ± 0.008	0.19 ± 0.009	0.40
Mg	0.020 ± 0.0010	0.017 ± 0.0007	0.06
Na	0.044 ± 0.0020	0.046 ± 0.0017	0.55
S	0.069 ± 0.0029	0.063 ± 0.0023	0.10
K	0.17 ± 0.0066	0.15 ± 0.0044	0.03
Trace minerals (ppm)			
Cu	0.35 ± 0.046	0.37 ± 0.044	0.90
Fe	1.83 ± 0.150	1.81 ± 0.181	0.98
Mn	1.14 ± 0.045	1.05 ± 0.035	0.12
Sr	3.35 ± 0.195	2.61 ± 0.181	0.001
Rb	1.11 ± 0.065	0.98 ± 0.044	0.10
Zn	11.4 ± 0.66	11.3 ± 0.99	0.96
GEFM (kcal/kg)	1609 ± 0.03	1582 ± 0.03	0.34
TDGE (kcal/day)	3642 ± 161.5	3922 ± 226,3	0.79
SCC (log 10/mL)	1.35 ± 0.277	1.63 ± 0.375	0.003

^1^ Provided by subcutaneous injections containing 0.83 mg of Cu per kg BW. ^2^ The experimental unit was the animal (*n* = 9 for Cu supplemented group and *n* = 8 for control group per lactation week). In addition, fat, protein, lactose, and dry matter concentrations were computed as the mean of the two aliquot replicates from each milking. ^3^ Statistical analyses considered only the effects of Cu supplementation and lactation week because their interaction was not significant for any traits.

**Table 3:** Effects of lactation week on body weight (BW) of hind and offspring, daily milk production (DMP), milk composition, gross energy content of fresh milk (GEFM), total daily gross energy content (TDGE), and somatic cell count (SCC) of Iberian red deer hinds fed a balanced diet. Data are means ± standard error of mean ^1^.

Item	2	4	6	10	14	18	*p*
Hind BW (kg)	106.4 ± 1.96	108.0 ± 2.06	108.9 ± 2.13	110.0 ± 2.12	109.6 ± 2.28	106.5 ± 2.04	0.73
Offspring BW (kg)	15.2 ^f^ ± 0.59	19.6 ^e^ ± 0.63	24.1 ^d^ ± 0.66	33.9 ^c^ ± 0.84	43.8 ^b^ ± 1.18	51.7 ^a^ ± 1.62	<0.001
DMP (mL/day)	3140 ^a^ ± 269.6	2832 ^a^ ± 263.7	2715 ^a^ ± 211.1	2098 ^b^ ± 139.2	2108 ^b^ ± 109.4	1582 ^b^ ± 132.5	<0.001
Milk composition							
Fat (%)	8.6 ^c^ ± 0.20	8.5 ^c^ ± 0.38	8.8 ^c^ ± 0.29	9.9 ^b^ ± 0.33	11.4 ^a^ ± 0.45	12.3 ^a^ ± 0.51	<0.001
Protein (%)	6.6 ^d^ ± 0.07	6.7 ^cd^ ± 0.13	6.7 ^cd^ ± 0.06	7.0 ^bc^ ± 0.09	7.2 ^b^ ± 0.11	7.7 ^a^ ± 0.16	<0.001
Protein:fat	0.78 ^ab^ ± 0.019	0.81 ^a^ ± 0.041	0.78 ^ab^ ± 0.028	0.72 ^bc^ ± 0.025	0.64 ^c^ ± 0.022	0.64 ^c^ ± 0.019	<0.001
Lactose (%)	4.1 ^b^ ± 0.06	4.3 ^ab^ ± 0.16	4.3 ^ab^ ± 0.08	4.6 ^a^ ± 0.07	4.3 ^ab^ ± 0.08	4.5 ^a^ ± 0.10	<0.001
Dry matter (%)	21.9 ^d^ ± 0.19	22.2 ^cd^ ± 0.38	22.8 ^cd^ ± 0.27	23.6 ^bc^ ± 0.33	24.8 ^ab^ ± 0.50	26.1 ^a^ ± 0.62	<0.001
GEFM (kcal/kg)	1462 ^d^ ± 0.02	1406 ^d^ ± 0.03	1486 ^d^ ± 0.02	1597 ^c^ ± 0.03	1735 ^b^ ± 0.04	1838 ^a^ ± 0.05	<0.001
TDGE (kcal/day)	4603 ^a^ ± 411.5	4146 ^ab^ ± 415.2	4033 ^ab^ ± 321.3	3346 ^bc^ ± 227.0	3641 ^bc^ ± 184.8	2873 ^c^ ± 237.6	0.005
SCC (log 10/mL)	1.64 ^a^ ± 0.336	1.46 ^a^ ± 0.476	1.58 ^a^ ± 0.386	1.31 ^c^ ± 0.282	1.34 ^c^ ± 0.252	1.53 ^b^ ± 0.270	0.02

^a–f^ Means within a row with different superscripts differ (*p* < 0.05). ^1^ The experimental unit was the animal (*n* = 17 per week). In addition, fat, protein, lactose, and dry matter concentrations were computed as the mean of the two aliquot replicates from each milking.

**Table 4:** Effects of lactation week on milk mineral composition of Iberian red deer hinds fed a balanced diet. Data are means ± standard error of mean ^1^.

Item	2	4	14	18	*p*
Macro minerals (%)					
Ca	0.28 ± 0.014	0.26 ± 0.017	0.23 ± 0.019	0.27 ± 0.018	0.21
P	0.18 ± 0.011	0.19 ± 0.009	0.22 ± 0.012	0.23 ± 0.012	0.08
Mg	0.019 ± 0.001	0.017 ± 0.001	0.017 ± 0.001	0.021 ± 0.001	0.05
Na	0.04 ± 0.002	0.04 ± 0.003	0.05 ± 0.003	0.05 ± 0.002	0.50
S	0.07 ± 0.003	0.06 ± 0.004	0.07 ± 0.004	0.07 ± 0.004	0.12
K	0.16 ± 0.007	0.17 ± 0.010	0.15 ± 0.007	0.15 ± 0.008	0.19
Trace minerals (ppm)					
Cu	0.47 ± 0.083	0.35 ± 0.051	0.31 ± 0.061	0.30 ± 0.036	0.21
Fe	1.42 ^b^ ± 0.143	2.34 ^a^ ± 0.256	1.43 ^b^ ± 0.154	2.01 ^ab^ ± 0.225	0.004
Mn	1.13 ^ab^ ± 0.044	1.02 ^b^ ± 0.055	1.03 ^b^ ± 0.058	1.27 ^a^ ± 0.053	0.010
Sr	3.65 ^a^ ± 0.293	3.05 ^ab^ ± 0.192	2.44 ^b^ ± 0.281	2.81 ^ab^ ± 0.300	0.003
Rb	0.98 ± 0.074	1.16 ± 0.090	1.04 ± 0.067	0.98 ± 0.080	0.33
Zn	11.6 ^ab^ ± 0.42	9.55 ^b^ ± 0.819	11.0 ^b^ ± 1.46	15.5 ^a^ ± 0.796	0.007

^a–b^ Means within a row with different superscripts differ (*p* < 0.05). ^1^ The experimental unit was the animal (*n* = 17 per week).
